# Bark and Wood Boring Insects—Past, Present, and the Future Knowledge We Need

**DOI:** 10.3390/insects12010028

**Published:** 2021-01-04

**Authors:** Dimitrios N. Avtzis, Ferenc Lakatos

**Affiliations:** 1 Agricultural Organization Demeter, Forest Research Institute, Hellenic, 57006 Vassilika, Greece; 2 Faculty of Forestry, University of Sopron, 9400 Sopron, Hungary

Bark and wood-boring insects represent a very diverse group of insects that includes bark and ambrosia beetles, cerambycids, weevils, jewel beetles, or even anobiids from the order of beetles (Coleoptera), but in the broader sense other insect orders like Lepidoptera (e.g., Cossidae), or Hymenoptera (e.g., Siricidae) may be included there too. Their ecological role in our forests and wooded lands varies from primary tree-killing habit to deadwood decomposers. Their preferred habitats include a great variety of terrestrial ecosystems from virgin forests to urban areas. Based on their significant role in our forests they were always at the forefront of various research activities, however, research priorities changed through time (e.g., for an overview on bark beetles see [1], or for a detailed review on *Ips typographus* [2]).

We may think that the knowledge acquired in the last decades on the biology, ecology, and evolution, monitoring, management, and control of bark and wood-boring insects covers already all aspects of these insects, but this is far from reality. New aspects and questions arise day by day. Our aim with this special issue is to make a comprehensive collection of the current knowledge on bark and wood boring insect species to set the direction for future research needs.

Few species make better use of and benefit more from the constantly increasing national and international trade than bark and woodboring insects worldwide [3]. Their ability to survive under bark or in the wood for longer periods renders these insects very difficult to intercept, while their reproductive strategy and trophic plasticity facilitate their rapid adaptation to new environments. A direct consequence of these traits is the frequent record of newly introduced bark and wood boring insect species, as recently in France, where eleven bark beetle and pinhole borers have been detected only in the short timeframe [4]. Even more alarmingly, wood-boring beetles have been detected in wooden transport boxes and pallets that were bought in museums [5]. Given that wooden crates and shelves are commonly used for storage, lending, and transportation between institutions, this finding highlights a frequently neglected risk. To mitigate the impact of bark and wood-boring insects, improved and novel approaches are constantly sought and investigated. For example, a series of recent studies has shown that trap color largely affects the beetle species trapped [6]. Brightly colored traps (yellow, green, and blue) attract a significantly higher number of flower-visiting longhorn beetles [7] than black ones. However, it is not only trap color that should be taken into account in a generic surveillance program. Recent experiments have shown the supremacy of multispecies lures over single-targeting lures. In particular, the addition of ethanol and α-pinene to bark beetle pheromones has increased the number of endemic bark and wood boring beetles in southeastern United States [8]. Something that had a similar effect at a larger scale, both on endemic and invasive Mediterranean pine bark beetle species [9]. As a recent study has concluded [10], a number of trapping variables, such as trap color, lure blend, and even height should be accordingly adjusted to maximize the efficacy of a surveillance program.

The impact of bark and wood-boring insects on forests are strongly amplified by the effects of climate change (e.g., weather extremes like storms, and hot and dry periods) that have become more frequent in the last decades. The current situation in the European Norway spruce stands is alarming. Damage caused by bark beetles (mainly by *Ips typographus* and *I. duplicatus*) in Germany, Switzerland, Austria, Cech Republic, and Slovakia among others, reached a higher level of magnitude than before [11]. Range expansion (sometimes even human “assisted” introductions), and changes in beetle’s ecology and behavior makes their management and control a real challenge. The walnut twig beetle, *Pityophthorus juglandis* is native to the southwestern USA and Mexico but in less than a decade, it has managed to expand northwards to other western and eastern States [12] and also invade Italy [13]. As a recent study has shown [14], *P. juglandis* captures in pheromone traps increased together with the mean weekly air temperature and decreased as the mean minimum relative humidity increased, evincing a strong dependency on the local temperature and rainfall regime. Indirectly, drought periods manipulate the interaction between carbon allocation and secondary metabolites (a component of plant defense against insects and pathogens) in plants [15], something that has largely defined the large-scale bark beetle outbreaks which have intensified in the last decades. In order to better comprehend the distribution of bark beetle attacks in relation to the ecophysiological traits of the host tree, the interaction between *Tomicus destruens* and *Pinus halepensis* was studied [16]. Interestingly, it was shown that at the plot level, *T. destruens* attacks are determined by the dispersal strategy of the beetle itself, rather than the physiology of the host tree, particularly during brood production. On the contrary, exotic ambrosia beetles of the genus *Xylosandrus* were strongly attracted by ethanol that is emitted from weakened trees [17], evincing a more selective strategy of these species, towards trees with disturbed physiology. However, the success of an infestation is not solely determined by the ecology and behavior of the beetles themselves, as in some cases, microorganisms provide an important associate to this target. The role of associated fungi for bark and wood-boring insects is well known [18,19]. Several ambrosia beetles became invasive in their introduced environment as a vector of various pathogenic fungi [20]. Moreover, it was found that a newly described bacteria species (*Pseudomonas typographi* sp. novel) not only assists in the nutrition of *Ips typographus* but also inhibits other fungal pathogens from infesting the bark beetle species [21]. An even more exquisite pattern was revealed in the *Picea abies-Armillaria-Ips* interaction [22]. Norway spruce trees weakened by drought and other abiotic factors are susceptible to *Armillaria* spp. infestation, that induces resin channels in wood. However, the excess of resin releases some volatile compounds that attract bark beetles, giving them the signal that this tree is suitable for colonization.

Understanding the complexity of interaction among trees, insects, and fungi and attempting to resolve the patterns observed, the Bark Beetle Mycobiome network has been recently established [23]. Studying these interactions will not only serve as a model in evolutionary biology but will also assist greatly in comprehending the mechanisms that underlie the epidemics that have now reached record proportions worldwide. To effectively accomplish that, the rigor of beetle identification should be facilitated either by curated DNA sequence databases [24] or even by enhanced and elaborated DNA protocols [25] in the case that a rapid identification is required (e.g., ports of entry). In any case, research in bark and wood-boring beetles is currently progressing and developing at a rapid pace, triggering changes and challenging our previous knowledge [26]. For that, the years to come will be even more exciting.

## Data Availability

Not applicable.
